# Advances on Antiviral Activity of *Morus* spp. Plant Extracts: Human Coronavirus and Virus-Related Respiratory Tract Infections in the Spotlight

**DOI:** 10.3390/molecules25081876

**Published:** 2020-04-18

**Authors:** Inès Thabti, Quentin Albert, Stéphanie Philippot, François Dupire, Brenda Westerhuis, Stéphane Fontanay, Arnaud Risler, Thomas Kassab, Walid Elfalleh, Ali Aferchichi, Mihayl Varbanov

**Affiliations:** 1 L2CM, Université de Lorraine, CNRS, F-54000 Nancy, France; thabtiines@yahoo.fr (I.T.); quentin.albert@univ-lorraine.fr (Q.A.); stephanie.philippot@univ-lorraine.fr (S.P.); francois.dupire@univ-lorraine.fr (F.D.); stephane.fontanay@univ-lyon1.fr (S.F.); arnaud.risler@univ-lorraine.fr (A.R.); thomas.kassab@me.com (T.K.); 2 Laboratoire d’Aridoculture et Cultures Oasiennes, Institut des régions Arides de Médenine, Route el Djorf, Médenine 4119, Tunisia; walid.elfalleh@fst.rnu.tn (W.E.); ferchichi.ali1@yahoo.fr (A.A.); 3 Department of Medical Microbiology, Academic Medical Center, Meibergdreef 15, 1105 AZ Amsterdam, The Netherlands; b.westerhuis@erasmusmc.nl; 4 Department of Viroscience, Erasmus MC, 3015 GD Rotterdam, The Netherlands; 5 INSA de Lyon, Université de Lyon, CNRS, UMR5240, F-69622 Villeurbanne, France; 6 Energy, Water, Environment and Process Laboratory, (LR18ES35), National Engineering School of Gabes, University of Gabes, Gabes 6072, Tunisia

**Keywords:** *Morus* spp., human coronavirus, respiratory viruses, picornaviruses, crude extract, antiviral activities

## Abstract

(1) Background: Viral respiratory infections cause life-threatening diseases in millions of people worldwide every year. Human coronavirus and several picornaviruses are responsible for worldwide epidemic outbreaks, thus representing a heavy burden to their hosts. In the absence of specific treatments for human viral infections, natural products offer an alternative in terms of innovative drug therapies. (2) Methods: We analyzed the antiviral properties of the leaves and stem bark of the mulberry tree (*Morus* spp.). We compared the antiviral activity of *Morus* spp. on enveloped and nonenveloped viral pathogens, such as human coronavirus (HCoV 229E) and different members of the *Picornaviridae* family—human poliovirus 1, human parechovirus 1 and 3, and human echovirus 11. The antiviral activity of 12 water and water–alcohol plant extracts of the leaves and stem bark of three different species of mulberry—*Morus alba* var. *alba*, *Morus alba* var. *rosa*, and *Morus rubra*—were evaluated. We also evaluated the antiviral activities of kuwanon G against HCoV-229E. (3) Results: Our results showed that several extracts reduced the viral titer and cytopathogenic effects (CPE). Leaves’ water-alcohol extracts exhibited maximum antiviral activity on human coronavirus, while stem bark and leaves’ water and water-alcohol extracts were the most effective on picornaviruses. (4) Conclusions: The analysis of the antiviral activities of *Morus* spp. offer promising applications in antiviral strategies.

## 1. Introduction

The last decades have seen the lack of new therapeutic drug developments against infectious diseases. Thus, emergence of several resistances and new epidemics are more and more noticed all over the world. These public health concerns have led to (re)evaluating traditional medical practices in order to highlight new molecules or new mechanisms of action. Plants like *Morus* spp., jackfruit (*Artocarpus heterophyllus*), *coffea,* and others are more and more studied in this context. Their potential activity against viral infections have been previously described in several studies [1,2,3,4].

Viral respiratory infections cause life-threatening diseases in millions of people worldwide every year. Human coronaviruses and several picornaviruses are responsible for regular worldwide epidemic outbreaks, thus representing a growing global public health problem. For instance, picornavirus infections are the most common infections identified in patients of all ages, and during peak season, picornaviruses cause 82% of all episodes of acute nasopharyngitis [5,6]. Among these viruses, enteroviruses like poliovirus 1 (PV1), human echoviruses, and parechoviruses share common features—they are small (20–30 nm), nonenveloped, positive single-stranded RNA viruses, causing mild respiratory diseases; however, HPeV type 1 and 3 (HPeV1 and 3) and echovirus 11 (Echo 11) can cause severe systemic diseases in young infants [7]. Structurally different, human coronaviruses (HCoVs) are bigger (120–160 nm), enveloped, positive single-stranded RNA viruses responsible for a large portion of upper and mild respiratory tract infections, like the common cold, bronchitis, or pneumonia. They have been isolated worldwide. Most established coronaviruses exhibit winter seasonality, with global geographical distributions of the documented cases [8]. They also can be severe and deadly for newborns, young infants, elderly, or immunosuppressed patients and can be associated with high rates of mortality. Indeed, no treatments, nor vaccines, are available against HCoVs, and the existing treatments are essentially symptomatic. Few new molecules show an interest in this context (i.e., glycyrrhizin and nelfivanir) [9,10,11].

The lack of specific treatment and the emergence of several and recent pandemics due to coronaviruses like SARS (severe acute respiratory syndrome, 2002–2003) and MERS (Middle East respiratory syndrome coronavirus, 2012), as the actual epidemic started in China (December, 2019), where a new HCoV is emerging (2019-nCov, then renamed SARS-CoV-2 [12]), underline the strong need of further investigations in the physiopathology and treatments of such pathologies [11,13].

The development of research fields like ethnopharmacology highlight the interest in traditional medical practices. In this context, *Morus* species have been reported for their medicinal uses in many regions of the world. Its antibacterial and antifungal activities have been previously studied [14,15,16]. The genus *Morus*, which belongs to the family *Moraceae*, counts 24 species with at least 100 varieties. The origins of *Morus spp*. are believed to be in Asia, but the plant has spread all over the world, and it can now grow from temperate to subtropical regions. Its growth does not require specific soil properties [17,18].

For centuries, Asian populations, especially Chinese populations, traditionally used *Morus spp*. against fever, sore throat, to protect the liver, enhance eyesight, rheumatism, diuresis, and blood pressure [18,19]. Consequently, the different parts of the plant have been studied for potential biological activities [14,15,18,19]. Several chemical families have been isolated in *Morus* species, like flavonoids, benzofurans, stilbene, polyhydroxylated alkaloids, and kuwanons, showing a large variety of pharmacological activities. For example, flavonoids and oxyresveratrol showed anti-inflammatory activities, while mulberrofuran showed antibacterial activities against pathogens like *Staphylococcus aureus*. The group of kuwanons (isoprenylated flavonoids) include various compounds like kuwanon C, E, G, H, J, and S, which exhibit distinct biological activities. Indeed, kuwanon G showed antibacterial activities against *Streptococcus mutans* and methicillin-resistant *Staphylococcus aureus* at 12 µg/mL (minimal inhibition concentration) [20]. Kuwanon C has shown an antifungal activity against *Candida albicans* and *Saccharomyces cerevisiae* [16].

Studies exploring the antiviral activities of *Morus* extracts are less numerous despite a confirmed potential. Indeed, mulberry juice reduces the cytopathogenic effect of murine norovirus 1 (MNV-1) and feline calicivirus FCV-F9 [15]. Arylbenzofurans extracted from *Morus* cortex are described like replication inhibitors of the hepatitis C virus (HCV) [15]. Scientific literature also describes kuwanons (among others chemical structures found in *Morus* spp.) as potentially antiviral. Indeed, the authors of [16] reported kuwanon H as a human immunodeficiency virus (HIV) inhibitor, leachianone as a human simplex virus 1 and 2 (HSV 1 and 2) inhibitor, and three flavonoids as influenza, respiratory syncytial virus (RSV), and adenoviruses inhibitors [1,4]. Other kuwanons, like kuwanon G, a prenylated flavon, have been studied for several biological properties, like anti-inflammation activity, anti-oxidant, and antibacterial activities [21]. Though the bioactivity of kuwanon G is evident, no antiviral activity has yet been described for this compound.

In this context, *Morus* species show a good pharmacological potential against several pathologies, including emerging viruses. Considering the needs of new therapeutic strategies against pathogenic respiratory viruses like coronaviruses and picornaviruses, this study proposes to evaluate the potential activity of aqueous and hydromethanolic extracts from the stem barks and leaves of three different species of mulberry—*Morus alba* var. *alba*, *Morus alba* var. *rosa*, and *Morus rubra* on human coronavirus 229E and four different members of the *Picornaviridae* family—human poliovirus 1, human parechovirus 1 and 3, and human echovirus 11. The study was further extended to the analysis of the phytochemistry profiles of the plants by gas chromatography coupled with mass spectrometry (GC–MS) and liquid chromatography coupled with mass spectrometry (LC–MS) and the quantitative investigation by GC–MS.

## 2. Results

### 2.1. Mass Spectrometry Characterisation of Morus spp. Extracts

In this study, we achieved the characterization of various compounds, like flavonoids and other phenolic components extracted from lyophilized mulberry leaves and stem barks, from water and hydroalcohol extracts. Identification of mulberry leaves and stem bark constituents was carried out on the basis of the complementary information obtained from high-resolution mass spectrometry (ESI/QTOF). To the best of our knowledge, the list of the identified compounds is consistent with the one reported in the literature in the three cultivars analyzed. Our HRMS analysis revealed the presence of different polyphenolic compounds in the extracts, with coumarins, tannins, triterpenes, and flavonoids being the major ones. We further identified specific compounds found in the majority and in the most effective antiviral extracts, such as alkaloids (1-deoxynojirimycin), prenylated flavonoids (kuwanon G), and stilbenoids (mulberroside A).

### 2.2. Cytotoxicity Assays

The results of in vitro cytotoxicity of the aqueous and hydromethanolic extracts are presented in Table 1. For hydromethanolic stem barks extracts, the CC50 ranged from 162.33 µg/mL (for *M. rubra* extract) to 253.33 µg/mL (for *M. alba* var. *alba* extract). For hydromethanolic leaves’ extracts, the CC50 ranged, respectively, from 908.20 µg/mL (for *M. alba* var. *rosea* extract) to 1051.66 µg/mL (for *M. rubra* extract). Aqueous stem barks’ extracts had CC50 ranged between 3166.00 µg/mL (for *M. alba* var. *rosea* extract) to 4330.33 µg/mL (for *M. alba* var. *alba* extract). Finally, leaves’ aqueous extracts of the three studied taxa had the same CC50 values, >5000 µg/mL.

Overall, hydromethanolic stem barks’ extracts were the most cytotoxic (mean = 197.77 ± 48.77 µg/mL), while aqueous leaves’ extracts are the less cytotoxic (>5000.00 µg/mL).

**Figure 1:**
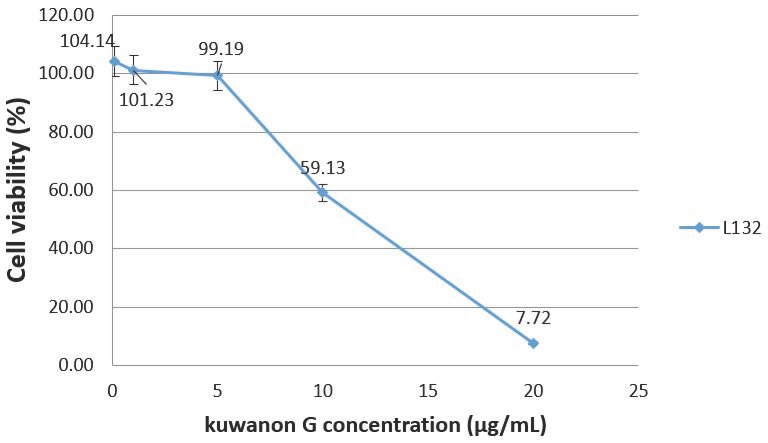
Cytotoxicity of kuwanon G on L-132 cell at 72 h post-treatment.

Figure 1 presents the cytotoxic effects of kuwanon G on L-132 cells and revealed that a concentration of 5 µg/mL does not induce a decrease in cell viability (99.19% compared to the control). However, cells viability decreases for higher concentrations and reaches 50% for a concentration estimated at 12 µg/mL.

### 2.3. Screening of Antiviral Activities

The antiviral activity of stem barks and leaves’ hydromethanolic and aqueous extracts are shown in Table 2.

Concerning the picornavirus (HPeV1, HPeV3, and Echo11) infections, no significant inhibition of the viral infection has been noticed at the tested concentration (data not shown). For example, aqueous and hydromethanolic stem bark extracts led to a viral titer (log_10_ CCID50/mL) from 4.96 (*M. rubra*) to 5.67 (*M. alba* var. *alba*) after PV1 infection, as shown in Table 2. The inhibition percentage ranged from 2.67 (*M. alba* var. *alba*) to 14.86 (*M. rubra*).

Considering HCoV 229E, the inhibition percentages of viral infectivity ranged from 34% to 36% for the aqueous stem bark extracts and from 37% to 45% for the hydromethanolic stem bark extracts. Hydromethanolic stem barks extracts of *M. alba* var *rosea* led to production of the lowest HCoV 229E titer of 1.98 and inhibited viral infectivity down to 45%. With aqueous extracts, the highest inhibition percentage is 36% for *M. alba* var. *rosea*.

Aqueous and hydromethanolic leaves’ extracts led to a viral titer (log_10_ CCID50/mL) ranging from 0.00 (*M. alba* var. *alba*) to 2.24 (*M. alba* var *rosea*). As for stem bark extracts, inhibition percentages of the hydromethanolic leaves’ extracts seem higher than the inhibition percentages of the aqueous extracts. Indeed, inhibition percentages of the hydromethanolic leaves’ extracts ranged from 67% (*M. rubra*) to 100% (*M. alba* var. *alba*) when inhibition percentages of the aqueous leaves’ extracts ranged from 38.54% (*M. alba* var *rosea*) to 48% (*M. alba* var. *alba*).

All the studied extracts were more active against coronavirus, which is an enveloped virus, than against nonenveloped picornaviruses.

**Figure 2:**
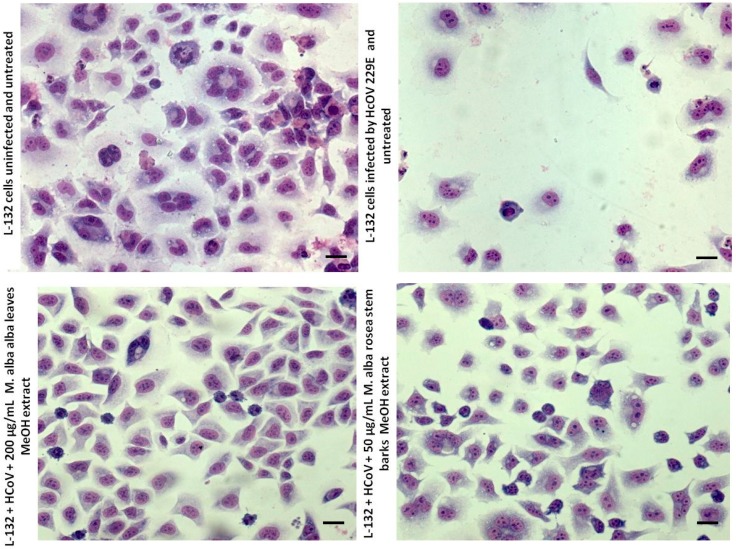
Human coronavirus (HCoV) 229E cytopathogenic effects observed with hydromethanolic extracts of *Morus alba* var. *alba* leaves and *Morus alba* var. *rosea* stem barks. Scale bar, 20 µm.

Thus, Figure 2 shows that the cytopathogenic effect (CPE) is strongly reduced after 200 µg/mL of leaves’ methanolic extract of *Morus alba* var. *alba* and after 50 µg/mL of steam barks’ methanolic extract of *Morus alba* var. *rosea*. These results are relevant with the calculated inhibition percentages.

### 2.4. Antiviral Activities of Kuwanon G

**Figure 3:**
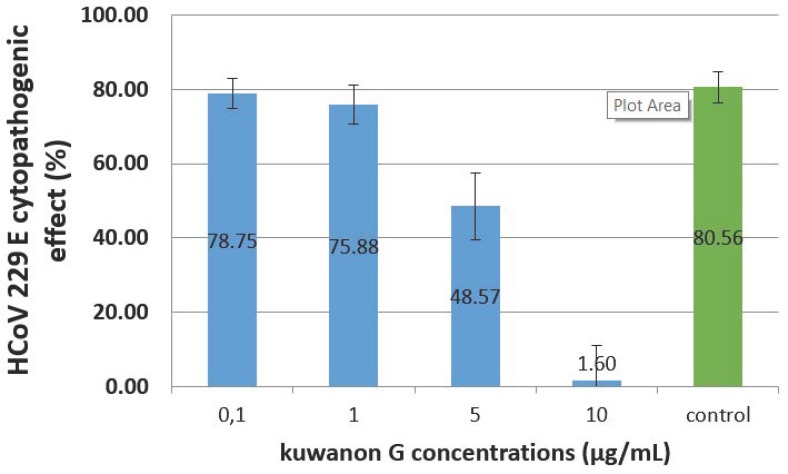
Evaluation of HCoV 229E cytopathogenic effects on L-132 cells (MOI = 1) in the presence of kuwanon G at different concentrations between 0.1 and 10 µg/mL at 72 h post-infection.

Given that all the *Morus* extracts were more active against the coronavirus, the antiviral activity of kuwanon G, present in the extracts, was evaluated on HCoV 229E. Figure 3 shows the CPE of HCoV 229E, after 72 h, on L-132 cells treated with several concentrations of kuwanon G. Concentrations of 0.1 and 1 µg/mL do not reduce the CPE, which remains respectively at 79% and 76% when the control (infected, nontreated cells) is 81%.

However, a concentration of 10 µg/mL strongly reduced the CPE down to 2%.

The drug concentrations needed to inhibit 50% of the viral CPE (EC50), as well as to induce 50% cell death (CC50), were determined in the 72-h assays. Kuwanon G antiviral activities (EC50) for the different MOI (0.01, 0.1, and 1) ranged from 0.11 ± 0.13 µg/mL to 0.56 ± 0.2 µg/mL and 5.61 ± 0.67 µg/mL, while the value obtained for CC50 was 9.45 ± 0.55 µg/mL. The resulting selectivity index (SI) (the ratio of CC50 to EC50) for kuwanon G was 86.73, 17.04, and 1.70, respectively (Figure S1).

## 3. Discussion

The ethnopharmacological approach has the potential to identify new antiviral compounds, yet relatively few antiviral screenings of plant ethnomedicines have been conducted.

The 50% cytotoxic concentrations (CC50) of studied extracts of the three *Morus* species were different between the aqueous and hydromethanolic extracts and between the leaves and stem barks’ extracts. The fact that methanolic extracts seem to be more toxic indicates that the organic fraction may contain more cytotoxic compounds. Further analyses of the extracts could provide more information about these compounds.

It also appears that extracts can exert antiviral effects in vivo, but this effect may not be detected by in vitro assays because of the extremely low concentrations of extracts tolerated by the cells in the artificial system [22]. It is also possible that the total extract contains synergistic compounds that are necessary to ensure an antiviral activity.

Our results suggest that hydromethanolic extracts are more efficient in inhibiting the HCoV 229E infection in L-132 cells.

Indeed, aqueous and hydromethanolic stem barks and leaves’ extracts showed lower antiviral activity towards picornaviruses compared to HCoV 229E at the tested concentrations. Certainly, hydromethanolic leaves’ extracts showed the best antiviral activity against enveloped viruses, especially the *M. alba* var. *alba* extract, which completely eliminated the HCoV 229E infection (Figure 2). *M. alba* var. *rosea* stem barks’ hydromethanolic extract was also the most active against HCoV 229E.

The better inhibitions against the enveloped virus (HCoV 229E) than the nonenveloped viruses (PV 1, HPeV 1, HPeV 3, and Echo 11) suggest that the active components might inhibit the interaction between the binding sites of the virus to the host cells by inhibiting a ligand of the viral envelope. The interaction between the virus envelope and the extracts could be related to the binding of phytochemical phenolic compounds with the protein coat of the virus and the viral attachment to the host cells.

In view of the significant proportion of plant extracts that have yielded positive results in these screenings, it seems reasonable to conclude that there are probably numerous types of antiviral compounds (like kuwanons, mulberrofuran, or oxyresveratrol) in these materials. Further characterization of the active ingredients of these plants might reveal more useful compounds [15,16].

A number of medicinal plants have been reported to contain compounds possessing strong antiviral activity [2]. It has been demonstrated that two prenylated flavonoids, namely leachinone G and mulberroside C, from seven known compounds isolated from *M. alba* root barks, were active principles for anti-HSV-1 activity, presenting 50% inhibitory concentrations of 1.6 μg/mL and 75.4 μg/mL, respectively [14]. Moracine P, moracine O, moracine M, and mulberrofurane K, 2-arylbenzofuran derivatives isolated from root barks’ ethyl acetate fraction, had a strong activity against the hepatitis C virus [15]. In previous works, phenolic acids and flavonol glycosides were identified in mulberry leaves [23]. Caffeic acid and chlorogenic acid were the major identified phenolic acids which were studied for their antiviral activities. Caffeic acid potentially inhibited the proliferation of HSV2 and adenovirus3 (ADV3) at, respectively, EC50 of 87.3 µg/mL and 14.2 µg/mL [24], whereas chlorogenic acid possessed the strongest anti-ADV-11 activity (EC50 = 13.3 µg/mL). Chlorogenic acid and caffeic acid were potential inhibitors of hepatitis B virus multiplication, reducing the number of viral particles in the serum by blocking DNA synthesis [25].

The study presented in [4] highlighted the antiviral activity of a *Morus rotunbiloba* infusion against the herpes simplex virus type 2 (HSV2), and they determined a 50% inhibitory concentration of 0.52 µg/mL. Methanolic extracts of some Thai medicinal plants have been reported to exhibit anti-HSV1 [3] and anti-HSV 2 [1] activities by inactivating the viral infections on the plaque-reduction assay.

Concerning kuwanon G, our results simultaneously show that a dose of 5 µg/mL is not toxic for L-132 cells (viability at 99.19%) when the same dose is able to reduce the HCoV 229E CPE from 80.56% to 48.57%.

According to the authors of [2], flavonoids were able to block the virus RNA synthesis, and the antiviral activity appeared to be associated to these compounds, and hydroxylation at the 3-position is apparently a prerequisite for antiviral activity. For example, quercetin reduced viral particle productions of herpes simplex virus type 1, poliovirus type 1, parainfluenza virus type 3, and respiratory syncytial virus [26].

The different molecules present in the extracts could act with synergy on different stages of the virus replication cycle (endocytosis, uncoating, replication, etc.). It could interact with the membrane viral glycoproteins (proposed as the binding site of some viruses) or with specific cell receptors, thereby blocking viral entry into the host cell or inhibiting viral replication [27].

The exploration of the antiviral mechanisms of *Morus* extracts and, particularly, of kuwanon G would need an in-depth molecular investigation like genome quantification, gene expression analysis, and protein expression. Further research on compounds like kuwanon G can result in a more potent inhibitor able to stop the newly emerged SARS-CoV-2 strain of HCoV.

## 4. Materials and Methods

### 4.1. Plant Material

*Morus alba* var. *alba*, *Morus alba* var. *rosea*, and *Morus rubra* leaves and stem bark were harvested during May 2011 from mulberry trees in Gabes Province (Southern Tunisia: 33°40′N, 10°15′E). The plant was identified and collected by Dr I. Thabti from the Arid Land Institute, Tunisia (Institut des Régions Arides: IRA), and voucher specimens (VS1-MAA2011, VS2-MAR2011, and VS3-MR2011) were deposited at the Herbarium of Arid Land Institute. Plant material was collected, immediately put in aluminum foil on ice and away from direct sunlight, and stored at −20 °C in the laboratory upon arrival. Then, the plant material was washed with distilled water and lyophilized. The dried materials were ground into powder with a blender and stored in an air-tight container at −20 °C until use.

### 4.2. Aqueous and Hydromethanolic Extracts Preparation

Approximately 3 g of lyophilized leaves and stem barks of *Morus alba* var. *alba*, *Morus alba* var. *rosea*, and *Morus rubra L.* were extracted with 100-mL water/methanol and 50/50 (MeOH 50%) in a water bath at 80 °C for 15 min. The same amount (3 g) of lyophilized leaves and stem barks were extracted with water. Respective MeOH 50% and aqueous phases were filtered and concentrated under vacuum to give the crude extracts, which were lyophilized and stored at −20°C prior to experimentation.

### 4.3. Mass Spectrometry Analysis

GC–MS identification of alkaloid extracts and a quantitative GC–MS analysis by using external standards (kuwanon G) were performed as previously reported by the authors of [28]. The quoted method was used to compare the contents of the different leaves and stem barks’ extracts of the three studied species of *Morus* spp.

For identification, the LC system consisted of an U3000-Dionex apparatus (Thermo Fisher Scientific, Waltham, MA, USA) with an injector comprising a 5-µL loop. Two microliters of sample were injected. The analytical column used was an HILIC mixed mode-1 (100 mm × 2.1 mm) and eluted at a flow rate of 200 µL/min using a gradient ranging from 98% solvent B to 80% solvent B in a time span of 40 min. Solvent A consisted of water with 10-mM ammonium acetate, and solvent B consisted of pure acetonitrile. The ESI-HRMS was a microTOFQ^TM^ (Bruker Daltonics, Bruker, Bremen, Germany) apparatus. The LC coupled with high-resolution mass spectrometry (ESI/QTOF, Bruker Daltonics, Bruker, Bremen, Germany) was used in order to determine the accuracy mass of compounds in the sample extracts. The identification of compounds was performed by comparing the measured molecular mass with the calculated values ± 10 mDa. The analysis was performed in positive mode, and the compound was confirmed by auto-MSMS experiment when the intensity was higher than 1 × 10^4^. This system was able to detect the compounds in amounts lower than 1 µg/mL. We used a compass for the otof series 1.7 software (Bruker Daltonics, Bruker, Bremen, Germany, 2013), including otof Control 3.4, for acquisition and DataAnalysis 4.2 for data processing.

To quantify, we used an LC Systeme Advance ^TM^ (Bruker Daltonics, Bruker, Bremen, Germany) coupled with an ESI-TQ Evoq ^TM^ (Bruker Daltonics, Bruker, Bremen, Germany). The analytical column used was an InfinityLab Poroshell 120 EC-C18 column (50 mm × 30 mm) and eluted at a flow rate of 800 µL/min, using a gradient ranging from 5% solvent B to 99% solvent B in a time span of 40 min. Solvent A consisted of water with 1% of formic acid, and solvent B consisted of pure acetonitrile. Kuwanon G was detected by MRM mode at 20 eV with one quantifying transition 693 > 203 and two qualifying transitions 693 > 355 and 693 > 421. LCMS control and data processing was performed by MS Workstation 8.2.1. (Bruker Daltonics, Bruker, Bremen, Germany, 2016).

### 4.4. Antiviral Activity

#### 4.4.1. Cytotoxicity Assay

The cytotoxicity of hydromethanolic and aqueous extracts of *Morus alba* var. *alba*, *Morus alba* var. *rosea*, and *Morus rubra* was evaluated in human embryonic pulmonary epithelial cells: L-132 cell line (ATCC CCL-5), according to the MTT assay, as proposed by the authors of [29]. The cells were grown in Eagle’s minimum essential medium (MEM) (Sigma-Aldrich, St Quentin Fallavier, France) supplemented with 10% of heat-inactivated fetal bovine serum (FBS) (Sigma-Aldrich, St Quentin Fallavier, France).

To test the biological activity, the dried, crude extracts were dissolved in MEM complemented with 2% heat-inactivated fetal bovine serum (FBS) and dimethyl sulfoxide 2% (DMSO, Sigma-Aldrich, St Quentin Fallavier, France) to concentrations of 50 µg/mL (stem bark extracts) or 200 µg/mL (leaves’ extracts).

The assays were performed in triplicate using 96-well flat-bottom tissue culture plates. One-hundred microliters of increasing concentrations from 9.76 to 5000 µg/mL of plant extracts were added to the monolayers of L-132 cells at 80% confluence. The plates were then incubated at 37 °C in a 5% CO_2_ atmosphere. After 72 h, the supernatants were removed, and the wells were washed with phosphate-buffered saline (PBS). Ten microliters of MTT (5 mg/mL, in PBS) were added to each well, and the plates were incubated for 4 h at 37 °C. Then, 100 µL of sodium dodecyl sulfate (SDS) (100 µg/mL, in PBS) were added to the wells to solubilize the MTT crystals. The plates were incubated for 4 h in 37 °C. The absorbance was read at 570 nm using a 96-well plate ELISA reader (MultiSkan GO, Thermo Scientific, Saint Herblain, France), and the 50% cytotoxic concentration (CC50) was then calculated as previously described [30].

To confirm the results obtained with the MTT assay, the monolayers were also observed microscopically for estimation of the CPE (i.e., rounding and other marked morphologic changes with respect to control cells).

#### 4.4.2. Viruses Production

Human coronavirus HCoV 229E (ATCC VR 740) and single-stranded RNA viral strains (HPEV-1, HPEV-3, PV1-Lsc/2ab, and Echo11) were propagated in the L-132 cell line (ATCC CCL-5).

For virus titration, the cells (10^4^ cells/well) grown in 96-well tissue culture plates were incubated for 72 h at 33 °C in a 5% CO_2_ atmosphere with serial 10-fold diluted virus suspensions. Then, infected wells were recorded. Viral titers and CCID 50 (50% cell culture infectious doses) were calculated using the Reed and Muench method [31]. All plates were observed daily for a cytopathic effect (CPE), and the estimation of the viral titer was made on the third day.

The virus titers (log10 CCID50/mL) were 3.65 for HCoV 229E and 5.82 for PV1 (the dilution of virus required for CCID50/mL lytic effect 50% of the inoculated cultures).

#### 4.4.3. Antiviral Activity Assay of the Extracts

The antiviral activity of mulberry aqueous and hydromethanolic leaves and stem barks’ extracts were evaluated in vitro by the CPE method using 96-well flat-bottom tissue culture plates and a crystal violet (CV) staining test. In these assays, the concentrations used were 50 µg/mL for aqueous or hydromethanolic stem barks’ extracts and 200 µg/mL for aqueous or hydromethanolic leaves’ extracts. These concentrations have been chosen according to the cytotoxic assay and are much lower than the determined CC50 on the L-132 cells.

The virus suspension produced as described above was diluted to 1:10 in extract solutions before infection.

Then, 100 µL/well of culture medium, containing each extract (at its respective concentration) supplemented with viral suspension, were added to confluent monolayer cells. Controls consisted of culture medium alone, culture medium with extract, and untreated noninfected cells. Furthermore, all tests were compared with plates consisting of untreated infected cells.

All assay plates were incubated at 33 °C in a 5% CO_2_ atmosphere incubator for 72 h. All wells were then observed and scored for viral-induced CPE under an inverted light microscope and by the CV method, as previously described, to evaluate cell viability [29]. Briefly, the cells were fixed for 5 min with 3.7% of paraformaldehyde, then stained for 30 min with 0.1% of crystal violet in 1% ethanol. Finally, monolayers were washed 2 times with PBS; then, quantification of the cells was obtained by an absorbance measurement (540 nm) after dissolution in 100 microliters of 50% methanol in PBS. Cytopathogenic effect was calculated as a percentage of infected cells compared to noninfected control cells.

The viral titer and the inhibition percentage of the viral infection were determined as follows:

Ivi = [(VTuc − VTtc)/VTtc] × 100(1)

with Ivi: inhibition percentage of the viral infection, VTuc: virus titer in the untreated control, and VTtc: virus titer in the treated control.

#### 4.4.4. Antiviral Activity Assay of Kuwanon G

Prior to the antiviral tests, the cytotoxicity of kuwanon G was evaluated as described in 4.4.1. The tested concentrations ranged from 0 to 20 µg/mL. The results were calculated after 3 independent experiments, and 4 replicates for each concentration were tested. The cells were then incubated for 24 h at 37 °C. On the day of infection, dilutions of kuwanon G (Biosynth^®^ carbosynth, CAS No 75629-19-5) from 0.1 to 10 µg/mL were prepared in the culture medium, containing 2% of FBS. These concentrations have been chosen according to the kuwanon G cytotoxicity assay. Cascade dilutions of the HCoV 229E virus were prepared for each kuwanon G dilution in order to test the virus at a multiplicity of infections (MOIs) equal at 1, 0.1, and 0.01, and wells were incubated with the virus/kuwanon G mix. Each test was reproduced in 8 identical wells. The plates were incubated for 72 h at 33 °C. The analysis of the wells was performed by microscopic observation of the cytopathogenic effect of HCoV 229E in the presence and absence of kuwanon G and after staining of the cell monolayers with crystal violet, as previously described.

### 4.5. Statistical Analysis

All tests were carried out in triplicate, and the results were presented as means ± SD. Statistical analysis was performed using Microsoft Excel 2010 (Microsoft Corp., Redmond, WA, USA, 2010) and GraphPAD Prism software (San Diego, CA, USA, 2007). A Wilcoxon test was performed to compare the data. Statistical significance was defined as *p* < 0.05.

## 5. Conclusions

Given the pressing need for new antiviral agents and the limitations of in vitro antiviral testing for such agents, the results of this screening were promising. The elucidation of the role of active constituents in the leaves and stem barks of *Morus spp*. may provide useful leads in the development of antiviral therapeutics. The results of the present investigation provide further evidence of the importance of ethnopharmacology as a guide to the screening for biologically active plants materials in the context of new viral emergencies and resistances.

## Figures and Tables

**Table 1:** In vitro cytotoxic activity of stem barks and leaves’ aqueous and hydromethanolic extracts on L-132 cells. Experiments were performed in triplicate, and the results are representative of three independent experiments.

Vegetal Material		Extract	CC_50_ (µg/mL) ± SD
			L-132
**Stem barks**	***M. alba* var. *alba***	MeOH	253.33 ± 14.57
		Aq	4330.33 ± 629.77
	***M. alba* var. *rosea***	MeOH	178.00 ± 11.00
		Aq	3166.00 ± 671.63
	***M. rubra***	MeOH	162.33 ± 8.50
		Aq	3192.00 ± 885.91
**Leaves**	***M. alba* var. *alba***	MeOH	1034.15 ± 225.83
		Aq	>5000
	***M. alba* var. *rosea***	MeOH	908.20 ± 66.53
		Aq	>5000
	***M. rubra***	MeOH	1051.66 ± 207.76
		Aq	>5000

MeOH: hydromethanolic extract; Aq: aqueous extract. Each value in the table is represented as mean ± SD (n = 3).

**Table 2:** Antiviral activities of stem barks and leaves’ aqueous and hydromethanolic extracts on HCoV 229E and PV1.

Vegetal Material		Extract	HCoV 229E		PV1	
			Viral Titer (log_10_)	Inhibition (%)	Viral Titer (log_10_)	Inhibition (%)
**Stem barks** **(50 µg/mL)**	***M. alba alba***	MeOH	2.16 ± 0.52	41	5.44 ± 0.10	7
		Aq	2.40 ± 0.67	35	5.67 ± 0.29	3
	***M. alba rosea***	MeOH	1.98 ± 0.29	45	5.10 ± 0.36	12
		Aq	2.50 ± 0.87	36	5.61 ± 0.19	4
	***M. rubra***	MeOH	2.30 ± 0.17	37	4.96 ± 0.48	15
		Aq	2.42 ± 0.54	34	5.62 ± 0.33	3
**Leaves** **(200 µg/mL)**	***M. alba alba***	MeOH	0	100	5.40 ± 0.17	7
		Aq	1.88 ± 0.67	48	5.61 ± 0.67	4
	***M. alba rosea***	MeOH	1.05 ± 0.59	71	5.44 ± 0.10	6
		Aq	2.24 ± 0.65	39	5. 46 ± 0.24	6
	***M. rubra***	MeOH	1.19 ± 0.60	67	5.12 ± 0.67	12
		Aq	2 ± 0.50	45	5.43 ± 0.23	7
**Negatif control**			3.65 ± 0.17	0	5.82 ± 0.39	0

MeOH: hydromethanolic extract; Aq: aqueous extract. Each value in the table is represented as mean ± SD (n = 3). The viral titer is expressed as mean log_10_ ± SD.

## Supplementary Materials

The following are available online. Figure S1: Normalized antiviral effects of kuwanon G on L-132 cells infected with HCoV 229E (2 independent experiments; 8 replicates).
