# Supplementary material for: Advances on Antiviral Activity of Morus spp. Plant Extracts: Human Coronavirus and Virus-Related Respiratory Tract Infections in the Spotlight
Source: Molecules. 2020 Apr 18;25(8):1876. doi: 10.3390/molecules25081876 (PMC7221944; doi:10.3390/molecules25081876)
Supplement: Supplementary file 1 [file molecules-25-01876-s001.pdf]

The Supp. Fig 1 represents the normalized reduction of the cytopathogenic effect of kuwanon G compared to a control of non-treated-infected cells (EC50). The EC50 has been calculated for each MOI and were 5.61 ± 0.67 µg/ml for MOI 1; 0.56 ± 0.2 µg/ml for MOI 0.1; and 0.11 ± 0.13 µg/ml for MOI 0.01.

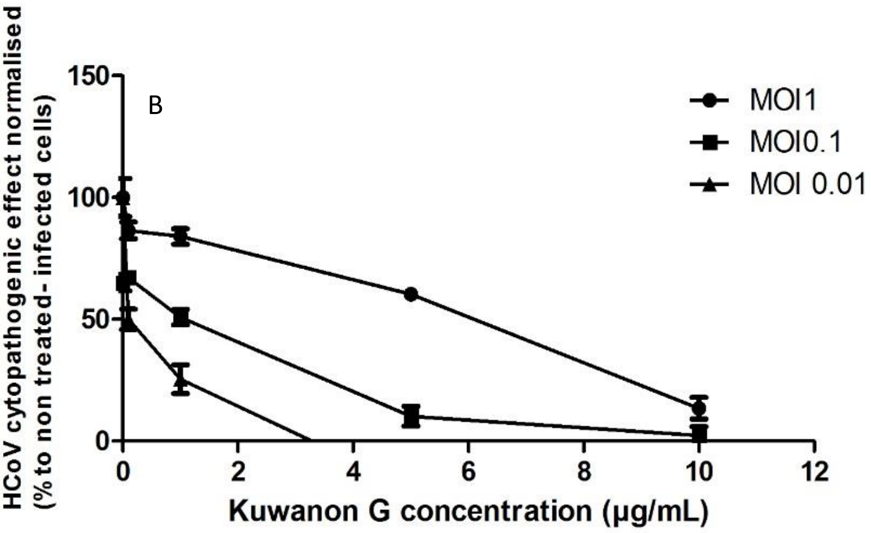

Supp. Fig 1: Normalized antiviral effect of kuwanon G on L-132 cells infected with HCoV 229E (2 independent experiments; 8 replicates).
